# The Enteric Fevers in the War

**Published:** 1916-01-29

**Authors:** 


### THE ENTERIC FEVERS IN THE WAR.

### A Triumph of Scientific Medicine.

UNTIL a few years ago the name of enteric fever was used by physicians as synonymous with typhoid fever, a circumstance which occasioned a good deal of confusion among the non-medical public. As matters have turned out, it has in the end been an advantage to possess two names for one and the same thing; for there are at the present day three closely allied diseases distinguishable which were formerly all included together. Enteric fever is a term now used to include all three of these diseases: the name typhoid fever is reserved for one of them, and the other two are called, respectively, paratyphoid A and paratyphoid B. The honour of reporting the first case of paratyphoid, as distinct from typhoid fever, belongs to Achard, of Paris, in 1896: it has since been identified in many other parts of the world, especially in the Tropics. Until the present campaign began, comparatively few cases of the paratyphoid fevers had been diagnosed in Great Britain or on the continent of Europe.

Typhoid fever is pre-eminently the scourge of field armies, especially when immobilised. The official returns of enteric—that is, as now classified, typhoid—fever in five recent campaigns are sufficient to prove this. In the Franco-Prussian War, the German Army reported 73,393 cases, with 6,965 deaths. In the Army of the Caucasus, Russo-Turkish War, there were 24,473 cases, with 8,900 deaths. In the American Army in Cuba there were 20,738 cases, with 1,580 deaths. The French Army in Tunisia had 4,200 cases, with 1,039 deaths—a death-rate of more than 5 per cent. of the whole army. In the Boer War we had 57,684 cases, with 8,022 deaths. These figures, it is quite certain, understate the ravages of enteric fever, for many of the milder cases went undiagnosed and unnotified.

### THE CHANCES OF AN EPIDEMIC.

The British Forces in France and Belgium have been engaged for more than a year in stationary operations. The terrain of their occupation is flat, and in many parts almost water-logged. The troops are badly over-crowded when they are in billets; and when they are in the trenches the question of sanitation is extremely difficult, for the trenches frequently become little better than sewers, strewn with half-buried corpses. Typhoid fever is rife in peace time among the civil population; and every circumstance favours a devastating outbreak of enteric fever. No such outbreak has taken place: about 400 cases of typhoid fever have been notified out of an army which cannot be far short of a million men. Moreover, the number of undiagnosed cases is certainly small, owing to the perfection of the arrangements for bacteriological investigation. No definite figures are available regarding the experience of the French and German Armies; but it is reported that both of them have suffered proportionally more than the British, though on nothing like the scale of previous campaigns.

The paratyphoid fevers have been more prevalent, it is true, than real typhoid. The figures given by Colonel Sir William Leishman at the Royal Society of Medicine show about 1,200 cases down to the end of October 1915. Whether there have been many undiagnosed cases is somewhat in dispute: Sir W. Leishman says not, but according to others there have been a good many. The point is of little importance, since these diseases are so much less severe than true typhoid that the mildest cases, those liable to escape diagnosis, last only a few days and are not deadly. Indeed, the fully developed disease (in France and Belgium) has caused but eighteen deaths in our Army.

### THE SYMPTOMS OF PARATYPHOID.

The symptoms of the paratyphoid fevers are a replica, almost to the minutest details, of those of true typhoid; but scaled down to about 25 per cent. of the latter's intensity. There are a very few minor differences, but these are so slight that a severe case of paratyphoid cannot be distinguished from a moderate case of typhoid save by bacteriological investigation; paratyphoid A can only be distinguished from paratyphoid B in the same way, for there is no difference clinically. The complications which may ensue are just as numerous, and of exactly the same nature, as those which are so familiar with typhoid fever; but they occur very much more rarely. The duration of the fever is, on the average, much shorter than that of typhoid, though there are wide limits in this respect: in nine-tenths of the cases the fever lasts three weeks or less, often much less; the extremes which the author has personally met with, in an experience of about 130 cases, have been five days and nine weeks —both were paratyphoid A.* In the British Expeditionary Force paratyphoid B has accounted for about 70 per cent. of the total cases of these two diseases, and paratyphoid A for most of the remainder; in a small percentage mixed infections have been reported. Before the war paratyphoid A had hardly ever been found in Western Europe, while paratyphoid B was rare in Asia and the Near East. When the returns of paratyphoid A were first made, it was thought that possibly the Indian troops, or British troops returned from India, had brought the infection with them; but investigation has apparently disproved this hypothesis. At Gallipoli there has been a larger outbreak than in France, chiefly of paratyphoid A. True typhoid has

* For further technical details about the paratyphoid fevers, the following papers may be consulted: Lieutenant-Colonel D. Harvey, *Journal of the R.A.M.C.*, June, July, August 1915; Captain Torrens and Lieutenant Wittington, *British Medical Journal*, November 13, 1915; Dr. H. Robinson, *Lancet*, October 16, 1915; *Proceedings of the Royal Society of Medicine*, Sir B. Dawson's paper, and subsequent discussion, November 1915; Dr. H. Wiltshire, *Practitioner*, December 1915.

also occurred there, but in very small numbers compared with the numbers of the paratyphoids.

### How the Troops are Protected.

Competent authorities ascribe this comparative immunity from enteric diseases in the British Expeditionary Force to two main causes: one, the extraordinary care taken by the R.A.M.C. to ensure correct hygiene and sanitation as far as possible in the very adverse conditions which prevail; the other, preventive inoculation. In the French and German armies the same precautionary measures are in force; but it may be doubted whether either is carried out with the same thoroughness and conscientiousness as by our R.A.M.C. Neither sanitation nor inoculation alone will suffice, but together they are highly successful. Into details of the sanitary problems and the measures adopted for dealing with them it is not necessary to enter. The subject is intricate and highly technical; and the Army bacteriologists have played an indispensable part in assisting the sanitary officers and other authorities. Preventive inoculation demands rather less cursory treatment and involves a brief excursion into bacteriology.

A good many years ago Eberth and other bacteriologists discovered a bacillus which was constantly associated with typhoid fever. This bacillus can often be obtained from the blood of patients in the early stages of the fever; it is frequently present in their excretions in later stages, and has also been found in their sweat and phlegm, and in the spots on the skin which are not uncommon. In fatal cases it can be demonstrated in the intestines, glands, spleen, liver, bile, and other situations. There is plenty of evidence that this bacillus is the sole direct cause of typhoid fever, and that it does not cause any other disease. It can be cultivated on suitable kinds of food in bacteriological laboratories, and it has many well-defined characters by which it can with certainty be identified. The name of this organism is the *Bacillus typhosus*, and it is often spoken of as Eberth's bacillus. There are also two other bacilli, known as paratyphosus A and paratyphosus B respectively, whose resemblance to each other and to Eberth's bacillus is extremely close. Under the microscope they all three appear exactly the same, and they behave in almost, but not quite, the same way to various tests. These three microbes are quite easily distinguishable from all others, but from each other only by the most delicate reactions in the hands of an expert bacteriologist. They are closely related, but the highest authorities declare that they are true species, not altered forms of one and the same bacillus: they breed true to type indefinitely.

### "Agglutination" Explained.

When these bacilli make good a lodgment in the living body and multiply in it, they produce poisonous substances, to which the symptoms of the host's illness are due. To neutralise these toxins the body manufactures protective substances. Whether there is more than one protective substance (against the toxins of each bacillus) is not certainly known; quite probably there may be several. There are also manufactured certain compounds, called agglutinins from the circumstance that their presence in a colony of the bacilli makes the latter adhere together in large clumps instead of moving freely and independently as they ordinarily do. Whether the agglutinins are the protective substances, or some of them, is uncertain; but as a working rule which is found to answer well the quantity of agglutinin is used as an index of the protectives found in the body. The reason why the agglutinins are thus used as a measure is that their amount can be estimated with accuracy, whereas that of the protective substances cannot. The agglutinins are sometimes spoken of as if they are the actual protective substances: they may be, but this is not absolutely proved. The estimation is carried out by mixing a given quantity of blood serum with a standard laboratory culture of bacilli, and noting how much of the former is required to "agglutinate" the latter: the process is called the Widal (or Widal-Gruber) test. It is further established that there are at least three separate agglutinins: one which agglutinates the *Bacillus typhosus*, but does not affect the paratyphosi; one which affects paratyphosus A alone; and one which confines its attentions to paratyphosus B.

For some years after an attack of typhoid fever the blood-serum of the patient will agglutinate more or less strongly the *Bacillus typhosus*; and experience shows that for a long time also the patient is practically immune against the infection of this disease, even in a district where cases of it abound. It is deduced that the agglutinating power of the blood-serum in a person not actually suffering from the disease is a fair measure of his resistance to infection. *Mutatis mutandis*, the same holds for the paratyphoid fevers, though possibly the duration of the agglutination period after the fever and the subsequent protection are shorter. Moreover, the agglutinating power of the serum in a patient actually suffering from any of these fevers rises rapidly as the disease progresses, and such a rise in a doubtful case of fever is evidence of typhoid or one of the paratyphoids as the case may be. Incidentally, a rapid rise of agglutinating power for *Bacillus typhosus* during an attack of typhoid fever is often accompanied by a much smaller rise of agglutinating power for the paratyphoid bacilli: this is termed the "group reaction." Paratyphoid infections display the same phenomenon.

### Protective Inoculation.

Now when a given number of killed typhoid bacilli, grown in a certain way and allowed to reach a certain age, are injected into a healthy individual, he suffers for a few hours from symptoms of mild ill-health; in an experience which runs into thousands of cases the writer has not met with any other ill-effects, nor with any medical officer who has seen any after such inoculation. In a few days the blood-serum exhibits the Widal reaction for *Bacillus typhosus*, but not for the paratyphoid bacilli. Theoretically, that is, the man is

(*Continued at foot of page* 387.)

### THE ENTERIC FEVERS IN THE WAR (*continued from page* 386).

protected against typhoid fever; and experience shows that his protection is actual as well as theoretical. In practice it is found that two injections, at an interval of one to three or four weeks, result in a greater production of agglutinins and a higher standard of protection than a single injection of the same total quantity of anti-typhoid "vaccine," and at the same time cause less inconvenience to the subject. The protection obtained wears off gradually: in a proportion of cases it disappears in two years, and those exposed to risk of infection are advised to have the inoculation repeated after the lapse of that period.

Not only is the inoculated person much less likely to suffer from typhoid fever than the uninoculated in the same circumstances; but also, if he does have it, the disease will be of much less severity. The statistics which support these statements are derived from all parts of the globe, and from the experience of many nations. In India up to 1906 there were every year from 1,000 to 2,000 cases among the British garrison, with a fatal result in about one case out of four. Since then the percentage of fully inoculated men has risen gradually to about 80 per cent. of the total, and typhoid fever has declined in frequency every year: by 1911 there were only 170 cases, with one fatal case in eight. In the case of twenty-four regiments which left England for India between 1904 and 1909 special returns were kept of the inoculated and uninoculated men. Out of 10,378 inoculated men 56 caught the fever, of whom 5 died; out of 8,936 uninoculated men, serving with the others, 272 contracted typhoid, of whom 46 died.

### Mixed Vaccines.

The latest development of protective inoculation against enteric fever is the suggestion that a mixed vaccine of typhosus, paratyphosus A, and paratyphosus B should be injected, in order to secure protection against all three diseases. Some authorities hold that this can be done. So far the Army Medical Service has not adopted it for fear that the use of a mixed vaccine might result in a smaller degree of protection against typhoid than now prevails. Protection against the much milder paratyphoid fevers is desirable in itself, but is not so urgent that the slightest risk is admissible of diminishing immunity against the far deadlier true typhoid.

### The Diagnosis of Enteric Fever.

From what has been said it will be realised that the blood-serum of a man suffering from any of the three enteric fevers "agglutinates" the bacillus of the particular kind which has invaded him. In doctors' phraseology, he has a "positive Widal reaction" for one or other of these fevers, and this reaction is largely used as a help in diagnosing enteric fever. It is true that the blood does not react thus until the patient has been ill for at least a week; but then the enteric fevers, especially typhoid, come on so gradually that it may be a week, or even two, before the sick man feels ill enough to consult a doctor. Further, the diagnosis of enteric fever may be extremely difficult in the early stages; so that the Widal reaction is of great value in assisting the physician to arrive at a conclusion. But in this campaign over 90 per cent. of the soldiers have been inoculated against typhoid. Opinions differ as to what proportions of such men have a positive Widal reaction; the estimates vary from 85 to 100 per cent. At any rate it is clear that the great majority of the men have already a positive reaction (for *Bacillus typhosus*, not for the paratyphoid bacilli); so that the value of this test in a soldier suspected of suffering from typhoid fever is seriously depreciated. Fortunately there are ways of surmounting this difficulty. If the Widal reaction be due, not to illness, but to previous inoculation, the intensity of it will remain at approximately the same level; whereas if the patient is suffering from typhoid fever, and the test be repeated at intervals of three or four days, it will be found that the intensity or degree of the agglutinating property of the blood-serum mounts steadily and rapidly as the fever progresses. In addition to this, advantage is taken of the fact that in the early weeks of typhoid fever the *Bacillus typhosus* is present in the circulating blood. By passing the needle of a small syringe into a vein of the arm, sufficient blood can be withdrawn for "cultivation" in the laboratory without causing any but the minutest degree of pain to the patient; after two or three days of incubation in a laboratory, it can be ascertained whether any typhoid bacilli are present. If they are, the diagnosis is established; but if not, that does not constitute proof that the patient has not got typhoid fever. The excretions can also be examined in the same way: typhoid bacilli are not present in them during the early stages of the fever, but towards the close of it, and even into convalescence, they can be detected in a large proportion of cases. Exactly the same principles apply to the diagnosis of the paratyphoid fevers, except that the men have not been inoculated against these, and therefore there is not the same element of doubt attaching to a Widal reaction for paratyphosus A or B.

### How the Bacilli are Identified.

In determining whether a bacillus of the enteric group is typhosus or one of its cousins, reliance is placed in the first instance on its behaviour towards various foods, or "culture media." Those most frequently used are certain sugars, which are fermented in slightly different ways by the three microbes. But this test alone is not regarded as absolutely conclusive: there is another, which is often called the Bordet-Durham reaction, and is the converse of the Widal reaction. In the latter, it may be recalled, the agglutinating power of a suspected person's blood-serum upon standard laboratory cultures of typhosus, paratyphosus A, or paratyphosus B is estimated, with a view to deciding whether the individual has in his blood the agglutinins of any of those three bacilli. In the Bordet-Durham test three sera, known to contain respectively the three agglutinins, are tested upon a suspected bacillus, isolated from a patient. If the bacillus regarded from its behaviour in sugar solutions as paratyphosus B, for instance, is agglutinated by serum known to agglutinate standard samples of paratyphosus B, then the identification of that organism is confirmed; if the suspected organism, however, is not agglutinated by the standard serum, doubt is cast upon its nature, and further investigations are undertaken before the bacteriologist will commit himself. In inoculated persons typhoid fever, on those occasions when it does occur, is extraordinarily mild, and therefore the more closely resembles the paratyphoid fevers: this complicates the task of diagnosis.

Statistics are not available as yet of the death-rates from typhoid fever among the inoculated and the uninoculated soldiers respectively. The only two fatalities in the writer's experience from this disease in the Expeditionary Force were both in uninoculated men. He has yet to meet with a fatal case of paratyphoid fever.

